# The effect of genetic polymorphisms on treatment duration following premolar extraction

**DOI:** 10.1038/s41598-021-94979-8

**Published:** 2021-08-05

**Authors:** Jiyon Yu, Yoon Jeong Choi, Sung-Hwan Choi, Han-Sung Jung, Ji Hyun Lee, Jung-Yul Cha

**Affiliations:** 1 Department of Orthodontics, Yonsei University College of Dentistry, Institute of Craniofacial Deformities, 50-1 Yonseiro, Seodaemun-gu, Seoul, 03722 Korea; 2 Division in Anatomy and Developmental Biology, Department of Oral Biology, Yonsei University College of Dentistry, Seodaemoon-gu, Seoul, Korea; 3 Department of Clinical Pharmacology and Therapeutics, Kyung Hee University College of Medicine, Dongdaemoon-gu, Seoul, 02453 Korea

**Keywords:** Genetics, Medical research

## Abstract

To elucidate genetic factors affecting orthodontic treatment duration, we employed targeted next-generation sequencing on DNA from the saliva of 117 patients undergoing orthodontic treatment after premolar extraction. The clinical characteristics of patients are summarized, and the association of clinical variables with treatment duration was assessed. Patients whose treatment duration deviated from the average were classified into an extreme long group or an extreme short group. We identified nine single nucleotide polymorphisms (SNPs) of six genes that significantly differed in the two groups via targeted sequencing. The frequency of the CC genotypes of *WNT3A*, *SPP1* (rs4754, rs9138), and *TNFSF11*, TT genotype of *SPP1* (rs1126616), and GG genotype of *SFRP2* was significantly higher in the extreme long group than in the short group. In the extreme short group, the TC genotype of *SPP1*, AA genotype of *P2RX7*, CT genotype of *TNFSF11*, and AG genotype of *TNFRSF11A* tended to exhibit higher frequency than in the long group. Taken together, we identified genetic polymorphisms related to treatment duration in Korean orthodontic patients undergoing premolar extraction. Our findings could lead to further studies predicting the prolongation of the orthodontic treatment duration, and will be of great aid to patients as well as orthodontists.

## Introduction

Treatment duration is one of the greatest concerns for orthodontists and their patients. Patients are often eager to know the treatment duration and how long they will require to use braces. Orthodontists sometimes change original treatment plans due to prolonged treatment durations. Further, if difficulties arise that complicate regular visits, such as emigration, studying abroad, military enlistment, or long-distance moving, the original treatment plans are often changed for short-term treatment methods. Timely completion of orthodontic treatment allows for accurate prediction of costs throughout the treatment period and improves treatment efficiency. Further, it can increase patient satisfaction and improve their quality of life. In contrast, prolonged treatment durations ‘burn-out’ the patients and reduce their cooperation, which inevitably worsens treatment outcome^1^. Therefore, the prediction of the orthodontic treatment period is of great importance for establishing the treatment plan and improving patient management for a better outcome.

Several studies have investigated variables that could influence treatment duration. These factors include age^2,3^, sex^2,4,5^, orthodontic appliances used^2^, magnitude of the orthodontic force^6^, molar relation^4^, the severity of malocclusion^7,8^, tooth extractions^2,4,5,8^, patient cooperation (e.g. missed appointments, breakage of appliances, wearing of elastics), and poor oral hygiene^9^. However, these reports had conflicting results with regard to the same variables. For example, some studies suggested that orthodontic treatment duration could be influenced by patient cooperation^2,4,10^, while others reported a low correlation between duration and cooperation^11^. Further, while some authors reported that initial severity of malocclusion, types of orthodontic appliances, and orthodontic force may affect treatment duration^4,5,7,8^, others rejected these claims^3,6^.

In addition, individual differences in relevant biological characteristics such as alveolar bone density, shape, and bone turnover rate may influence orthodontic tooth movement and thus affect the treatment period^12^. Alveolar bone metabolism is closely related to the speed of orthodontic tooth movement, resulting in different bone turnover rates under orthodontic forces based on patient-specific traits^13,14^. In an experimental study on beagle dogs, the group whose bone density was increased by promoting bone formation exhibited significantly reduced tooth movement speed compared to the control group, indicating that the quality of alveolar bone affected orthodontic treatment duration^15^.

These individual differences in physiological characteristics stem from respective genetic differences attributed to gene polymorphisms that lead to varied gene expression levels between individuals. In other words, genetic polymorphisms may contribute to differences in orthodontic tooth movement treatment duration. Multiple genetic polymorphisms have been related to orthodontic treatment duration, with studies reporting single nucleotide polymorphisms (SNPs) that affect tooth movement. It was reported that the polymorphism of *IL-1*, which encodes an inflammatory cytokine, initially discovered around alveolar bone, affects the speed of tooth movement^16,17^. In addition, genetic polymorphisms of *RANK*, *RANKL*, *OPG*, *COX2*, and *IL-6* have been reported to influence their expression levels in periodontal ligament fibroblasts, thereby affecting orthodontic tooth movement^18^. Furthermore, an SNP of the *SPP1* gene, encoding osteopontin (OPN), was reported to alter bone metabolism in tissues around teeth, subsequently affecting tooth movement and increasing root resorption^19^.

Despite the importance of genetic factors affecting orthodontic treatment duration, studies on specific genes and polymorphisms that influence tooth movement speed and treatment duration are lacking. In the current study, we hypothesized that genetic polymorphisms affect orthodontic treatment duration due to the resulting individual differences in physiological traits that influence tooth movement. In particular, these physiological differences are to some extent determined by genetic polymorphisms. Thus, we employed bioinformatics to identify genes that influence orthodontic treatment duration in Korean patients who underwent tooth extraction. In addition, genetic polymorphisms related to treatment duration were identified via targeted gene sequencing.

## Results

### Correlation between clinical parameters and orthodontic treatment duration

Among the clinical parameters of patients, horizontal anterior retraction was positively correlated (Beta = 0.1026, *P*-value = 0.0014) with treatment duration, while crowding and displacement exhibited a negative correlation (Beta = − 0.1241, *P*-value = 0.0029; Beta = − 0.1104, *P*-value = 0.0041, respectively). Age, sex, Angle classification, overjet, overbite, A point-nasion-B point (ANB), peer assessment rating (PAR) score, Frankfurt mandibular plane angle (FMA), and U1 to SN did not show any significant association. Multivariate linear regression was performed after adding all clinical variables as covariates and the analysis indicated horizontal anterior retraction as the variable most strongly correlated with orthodontic treatment duration (Table 1).

**Table 1:** Clinical parameters associated with orthodontic treatment duration.

Clinical parameters	Univariate analysis		Multivariate analysis	
	Beta (95% CI)	P value	Beta (95% CI)	P value
Age	0.1090 (− 0.0358 to 0.2537)	0.1387		
Gender	0.0004 (− 0.0090 to 0.0099)	0.928		
Angle classification	0.0008 (− 0.0150 to 0.0165)	0.9247		
Horizontal anterior retraction (mm)	0.1026 (0.0406 to 0.1646)	**0.0014**	0.5889 (0.0102 to 1.1676)	**0.0485**
Crowding (mm)	− 0.1241 (− 0.2049 to − 0.0433)	**0.0029**	− 0.3441 (− 1.4399 to 0.7517)	0.5394
Displacement (mm)	− 0.1104 (− 0.1852 to − 0.0357)	**0.0041**	− 0.0299 (− 1.2154 to 1.1556)	0.9607
Overjet (mm)	− 0.0217 (− 0.0749 to 0.0316)	0.4215		
Overbite (mm)	0.0244 (− 0.0236 to 0.0724)	0.3158		
ANB (T1)	0.0120 (− 0.0445 to 0.0685)	0.6751		
ANB (T2)	0.0078 (− 0.0500 to 0.0657)	0.7887		
PAR (unweighted)	− 0.0565 (− 0.1181 to 0.0052)	0.0725		
PAR (weighted)	− 0.1054 (− 0.3368 to 0.1259)	0.3687		
FMA (T1)	− 0.0708 (− 0.1985 to 0.057)	0.2748		
FMA (T2)	− 0.1105 (− 0.3126 to 0.0915)	0.2809		
U1 to SN (T1)	0.0497 (− 0.1146 to 0.2139)	0.5504		
U1 to SN (T2)	− 0.0072 (− 0.15 to 0.1356)	0.9207		

Simple (univariate) and multivariate linear regression analysis was used.

Bold values denote statistical significance at the P < 0.05 level.

*CI* Confidence interval, *Beta* Beta coefficient.

### Classification of extreme phenotype groups

The subjects were classified based on treatment duration. After creating a correlation plot between horizontal anterior retraction and orthodontic treatment duration, values outside the standard residual ± 1 were set as the extreme groups. Values above the standard residual (+ 1) were classified into the ‘extreme long group’ with a treatment duration longer than the average, and values below the standard residual (− 1) were classified into ‘extreme short group’ with a treatment duration shorter than the average. The number of patients in each group was 18.

### Clinical characteristics of the subjects

The clinical characteristics of the subjects are shown in Table 2. There was no significant difference between groups with regard to age, gender, Angle classification, crowding, displacement, overjet, overbite, ANB, FMA, U1 to SN, as well as PAR score. As expected, treatment duration was significantly different between groups, as they had been classified based on orthodontic treatment duration (Table 2).

**Table 2:** The clinical characteristics of patients.

Clinical variables	All patient (n = 117)	Extreme short group (n = 18)	Extreme long group (n = 18)	P value* Short vs long)
Age (years), median (range)	19.8 (12–46.9)	18.4 (12–34.3)	19.5 (12–26.7)	0.558
**Gender, n (%)**				
Female	93 (79.5)	15 (83.3)	13 (72.2)	0.423
Male	24 (20.5)	3 (16.7)	5 (27.8)	
**Angle classification, n (%)**				
Class I	44 (37.6)	7 (38.9)	7 (38.9)	0.073
Class II	57 (48.7)	10 (55.6)	5 (27.8)	
Class III	16 (13.7)	1 (5.5)	6 (33.3)	
Horizontal anterior retraction(mm), mean ± SD	4.45 (2.79)	4.07 (2.82)	3.99 (2.77)	0.934
Crowding (mm), mean ± SD	3.72 (3.61)	5.14 (3.45)	4.16 (3.88)	0.424
Displacement (mm),	3.97 (3.33)	5.14 (3.45)	4.84 (2.91)	0.780
Overjet (mm)	3.26 (2.29)	3.36 (2.01)	2.35 (2.11)	0.150
Overbite (mm)	1.57 (2.07)	0.53 (2.72)	1.36 (1.79)	0.285
ANB (T1)	4.01 (2.43)	4.57 (2.05)	3.43 (3.29)	0.220
ANB (T2)	3.66 (2.49)	4.25 (1.72)	3.08 (3.49)	0.212
FMA (T1)	29.90 (5.52)	31.17 (6.06)	28.66 (6.70)	0.246
FMA (T2)	30.24 (8.73)	30.21 (5.60)	28.61 (6.58)	0.436
U1 to SN (T1)	107.16 (7.07)	106.08 (8.05)	106.94 (9.52)	0.771
U1 to SN (T2)	101.00 (6.13)	100.41 (6.13)	102.13 (6.98)	0.437
PAR (unweighted)	5.22 (2.69)	6.00 (2.47)	6.17 (3.31)	0.865
PAR (weighted)	15.75 (9.97)	17.50 (9.95)	18.67 (12.42)	0.758
Treatment duration	26.59 (7.94)	15.67 (4.78)	39.06 (5.06)	**< 0.001**

Bold values denote statistical significance at the P < 0.05 level.

*Chi-square test or t test were used where appropriate.

### Association between SNPs and orthodontic treatment duration

Targeted capture sequencing identified 142 variants with a minor allele frequency of > 3% in the candidate regions. No common variants were significantly associated with orthodontic treatment duration after Bonferroni correction for multiple testing (cut-off *P*-value = 0.05/142 = 3.52 × 10^–4^). Only nine SNPs showed nominal significance (P < 0.05) in frequency between the extreme short and long groups (Table 3).

**Table 3:** SNPs significantly associated with orthodontic treatment duration (leveling and retraction period) in targeted sequencing study.

Gene (rs no.)	*Base change (AA change)	Geno-type	Group. No. (%)		**P value [Beta (95% CI)]				
			Extreme short group	Extreme long group	Dominant	Recessive	Codominant		Allele
							Hetero	Homo	
*WNT3A (rs752107)*	c.*185T > C	T/T	2 (11.1)	0 (0)	0.858 [7692.2 (0–2.957)]	**0.027** [10.076 (1.3–78.375)]	0.829 [573.38 (0–6.5082)]	0.777 [4175 (0–4.6521)]	**0.016** [6.529 (1.424–29.93)]
		T/C	6 (33.3)	3 (16.7)					
		C/C	10 (55.6)	15 (83.3)					
*SPP1 (rs4754)*	c.321T > C (p.D107D)	T/T	1 (5.6)	1 (5.6)	0.959 [0.927 (0.051–16.71)]	**0.018** [8.935 (1.455–54.88)]	0.45 [0.305 (0.014–6.643)]	0.45 [3.316 (0.148–74.47)]	0.069 [2.902 (0.919–9.17)]
		T/C	11 (61.1)	5 (27.8)					
		C/C	6 (33.3)	12 (66.7)					
*SPP1 (rs1126616)*	c.789C > T (p.A263A)	C/C	1 (5.6)	1 (5.6)	0.959 [0.927 (0.051–16.71)]	**0.018** [8.935 (1.455–54.88)]	0.45 [0.305 (0.014–6.643)]	0.45 [3.316 (0.148–74.47)]	0.069 [2.902 (0.919–9.17)]
		C/T	11 (61.1)	5 (27.8)					
		T/T	6 (33.3)	12 (66.7)					
*SPP1 (rs9138)*	c.*294A > C	A/A	1 (5.6)	1 (5.6)	0.959 [0.927 (0.051–16.71)]	**0.018** [8.935 (1.455–54.88)]	0.45 [0.305 (0.014–6.643)]	0.45 [3.316 (0.148–74.47)]	0.069 [2.902 (0.919–9.17)]
		A/C	11 (61.1)	5 (27.8)					
		C/C	6 (33.3)	12 (66.7)					
*SFRP2 (rs3810765)*	c.502 + 6C > T	G/G	6 (33.3)	11 (61.1)	0.085 [0.287 (0.07–1.187)]	0.699 [0 (0–2.968)]	0.654 [0.698 (0.146–3.35)]	0.606 [0 (0–11,428)]	**0.007** [0.212 (0.068–0.659)]
		G/A	7 (38.9)	7 (38.9)					
		A/A	5 (27.8)	0 (0)					
*P2RX7 (rs3751143)*	c.1487A > C (p.Q496A)	A/A	14 (77.8)	8 (44.4)	0.038 [4.968 (1.094–22.56)]	**0.038** [4.968 (1.295–78.38)]	**0.038** [4.968 (1.094–22.56)]	–	0.07 [3.309 (0.906–12.09)]
		A/C	4 (22.2)	10 (55.6)					
		C/C	0 (0)	0 (0)					
*TNFSF11 (rs12585229)*	c.388-3222C > T	C/C	4 (22.2)	11 (61.1)	**0.023** [0.17 (0.037–0.781)]	0.516 [0.513 (0.069–3.837)]	**0.028** [0.162 (0.032–0.819)]	0.153 [0.197 (0.021–1.829)]	**0.046** [0.35 (0.125–0.983)]
		C/T	11 (61.1)	5 (27.8)					
		T/T	3 (16.7)	2 (11.1)					
*TNFSF11 (rs931273)*	c.533-2050C > T	C/C	4 (22.2)	11 (61.1)	**0.013** [0.145 (0.032–0.66)]	0.516 [0.513 (0.069–3.837)]	**0.015** [0.127 (0.024–0.667)]	0.147 [0.2 (0.023–1.761)]	**0.029** [0.311 (0.109–0.886)]
		C/T	11 (61.1)	5 (27.8)					
		T/T	3 (16.7)	2 (11.1)					
*TNFRSF11A (rs4524034)*	c.616 + 726A > G	A/A	2 (11.1)	7 (38.9)	0.053 [0.167 (0.027–1.023)]	0.552 [1.687 (0.3–9.48)]	**0.032** [0.128 (0.02–0.834)]	0.405 [0.382 (0.04–3.682)]	0.357 [0.632 (0.238–1.676)]
		A/G	13 (72.2)	6 (33.3)					
		G/G	3 (16.7)	5 (27.8)					

*CI* Confidence interval, *Beta* Beta coefficient.

P value was adjusted for gender and age at the beginning of treatment. Bold values denote statistical significance at the P < 0.05 level.

*Nucleotide location number was assigned according to the *WNT3A* (Transcript ID: NM_033131.3), *SPP1* (Transcript ID: NM_001251830.1), *SFRP2* (Transcript ID: NM_003013.2), *P2RX7* (Transcript ID: NM_002562.5), *TNFSF11* (Transcript ID: NM_003701.3) and *TNSFRSF11A* (Transcript ID: NM_003839.3) mRNA sequence. Minor allele sequence is underlined in each position.

**Dominant and recessive model of genetic association study was based on alternative allele.

In the extreme long group, the frequency of the CC genotypes of *WNT3A*, *SPP1* (rs4754, rs9138), and *TNFSF11* (rs12585229, rs931273), the TT genotype of *SPP1* (rs1126616), as well as the GG genotype of *SFRP2*, was significantly higher than that in the short group. In contrast, the frequency of the TC genotype of *SPP1* (rs4754 and rs1126616), the AC genotype of *SPP1* (rs9138), the AA genotype of *P2RX7*, and the CT genotype of *TNFSF11* was higher in the extreme short group when compared with that in the extreme long group. Furthermore, the heterozygote of *TNFRSF11A* (AG genotype) showed overdominance in the extreme short group.

Three SNPs of *SPP1* (rs4754, rs1126616, rs9138) were linked together and rs9138, as an effective SNP, remained statistically significant after haplotype analysis was performed. The SNPs of *TNFSF11* (rs12585229, rs931273) were also linked together. Rs752107 of *WNT3A* and rs9138 of *SPP1* were identified as 3′-UTR SNPs. The rs3751143 SNP of *P2RX7* was a missense variant, while the SNPs of *TNFSF11* (rs12585229, rs931273) and *TNFSF11A* (rs4524034) were intron variants. The rs3810765 SNP of *SFRP2* was located in a splicing region involved in transcription. Remaining SNPs were synonymous variants encoding the same amino acid sequences.

**Figure 1:**
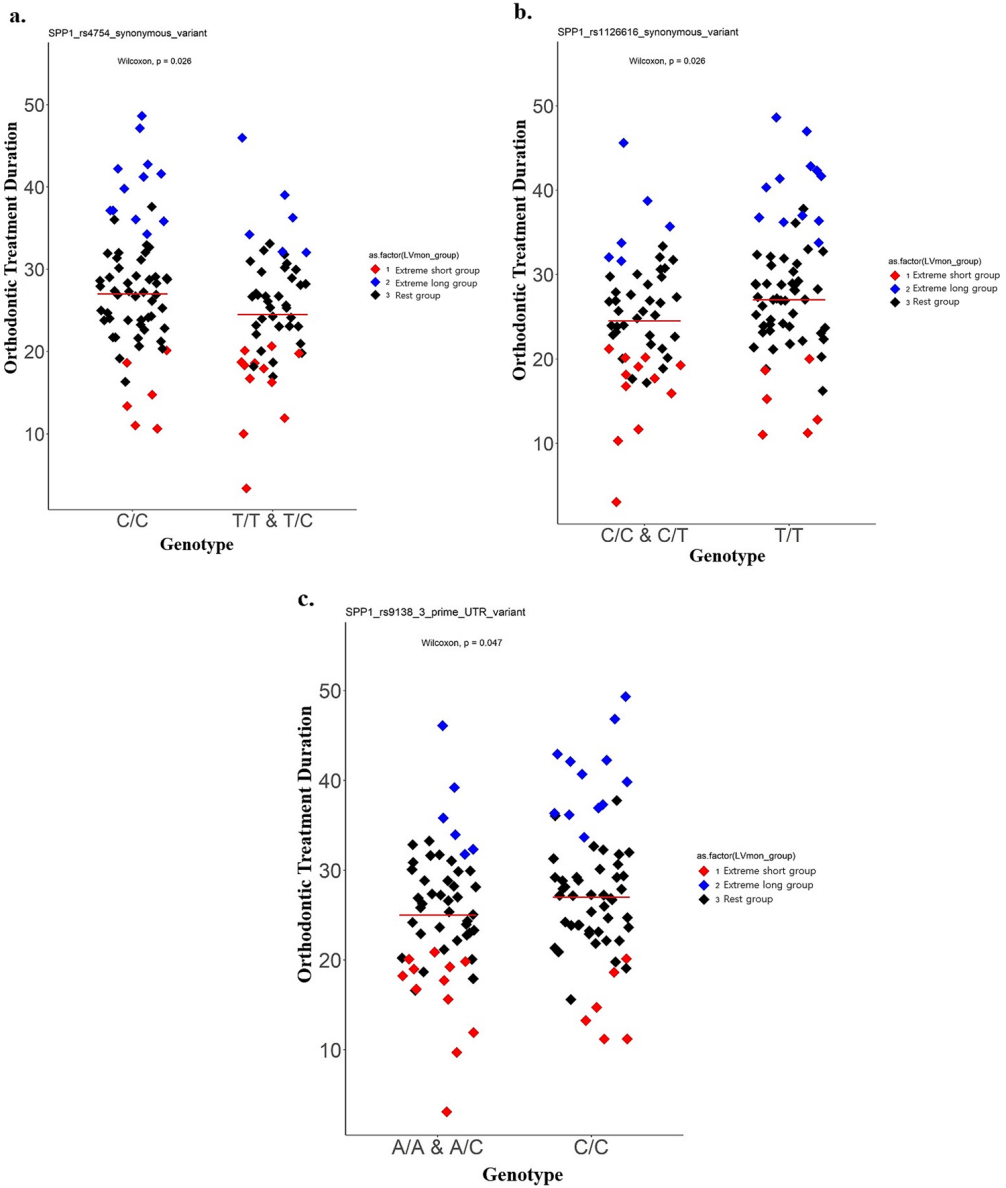
Orthodontic treatment duration according to genotypes in SNPs of *SPP1.* (**a**) *SPP1* (rs4754), (**b**) *SPP1* (rs1126616), (**c**) *SPP1* (rs9138), A scatter plot illustrating the orthodontic treatment duration in each genotype. The y-axis represents the orthodontic treatment duration values (months) and horizontal bars in the scatter plots represent mean values. Statistical analysis was performed with the Wilcoxon test. *P < 0.05.

**Figure 2:**
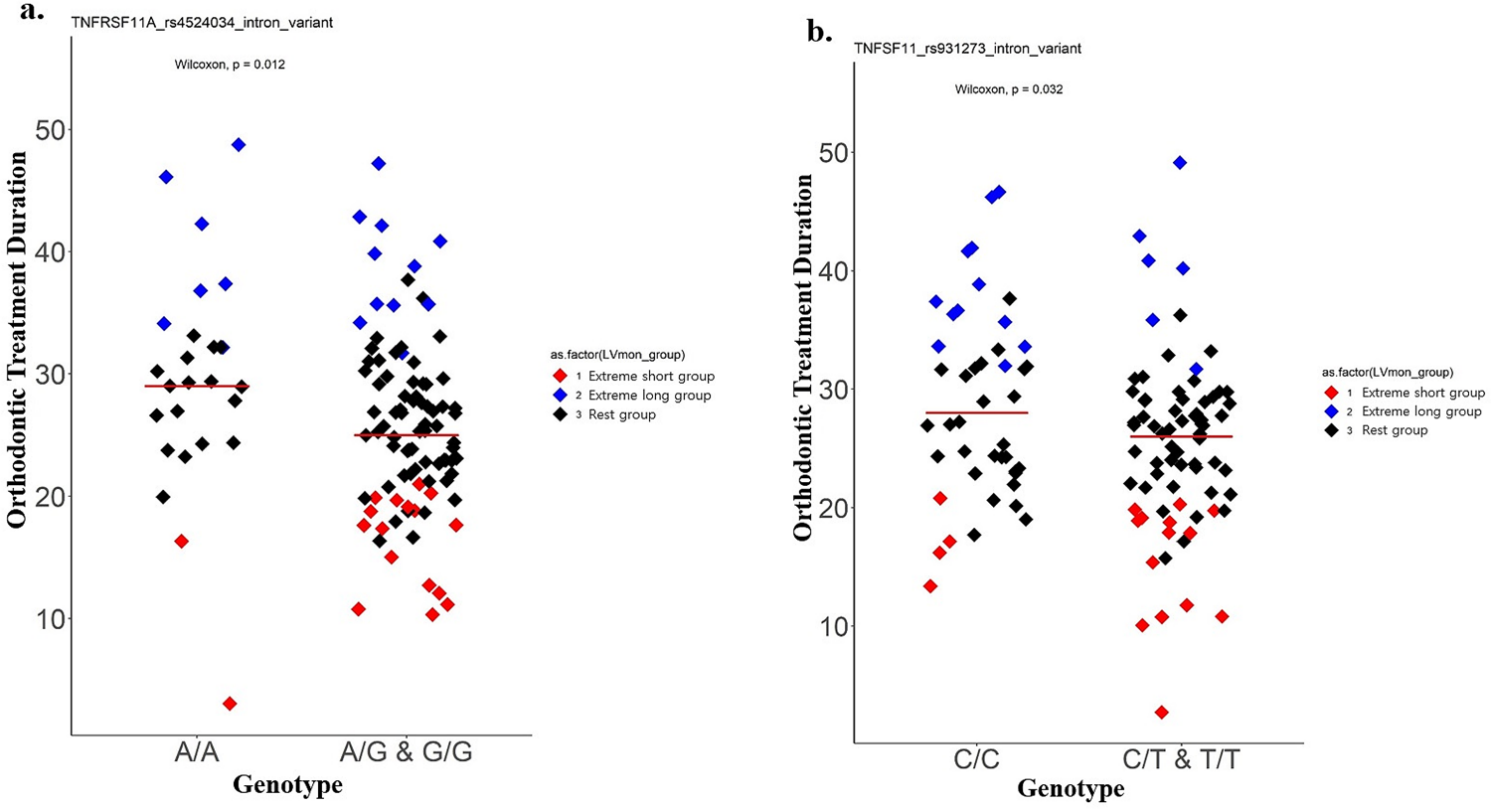
Orthodontic treatment duration according to genotypes in SNPs of *TNFRSF11A* and *TNFSF11.* (**a**) TNFRSF11A (rs4524034), (**b**) TNFSF11 (rs931273), A scatter plot illustrating the orthodontic treatment duration in each genotype. The y-axis represents the orthodontic treatment duration values (months) and horizontal bars in the scatter plots represent mean values. Statistical analysis was performed with the Wilcoxon test. *P < 0.05.

In all subjects, genetic association with orthodontic treatment duration for significant SNPs of extreme phenotype sampling study was showed on Supplementary Table 1. The SNPs significantly related to the treatment duration are represented using scatter plots. The CC type of *SPP1* (rs4754, rs9138) and the TT type of *SPP1* (rs1126616) were associated with longer orthodontic treatment duration when compared to other types (Fig. 1). The AA type of *TNFRSF11A* (rs4524034) and the CC type of *TNFSF11* (rs931273) were associated with an increase in treatment duration (Fig. 2). Other SNPs did not exhibit any significant difference in orthodontic treatment duration based on genotypes in all subjects (P > 0.05).

The SNPs discovered above and the clinical variables related to the orthodontic treatment duration in the univariate analysis were added as parameters for multivariate analysis to confirm the effect of clinical variables and genetic polymorphisms on the treatment duration. As a result, it was found that rs9138 of *SPP1* and rs4524034 of *TNFRSF11A* affect the orthodontic treatment duration (Supplementary Table 2). For more details, genetic information of significant SNPs in extreme phenotype sampling study and allele frequency in all subjects were described in Supplementary Table 3.

## Discussion

In the current work, we explored factors that influence orthodontic treatment duration, with a focus on genetic traits. Univariate linear regression was employed to analyze the clinical parameters of subjects, and horizontal anterior retraction exhibited a positive correlation with treatment duration. In other words, the greater the horizontal retraction of anterior teeth, the longer the treatment duration. In contrast, crowding was negatively associated with orthodontic treatment duration. Crowding was previously reported to influence the increase in leveling duration^9^. However, after the completion of leveling, crowding is resolved, and the extraction space decreases, leaving less available space for anterior tooth retraction. According to simple linear regression data on the degree of crowding related to leveling and retraction period (data not shown), the decrease in anterior retraction duration was greater than the increase in leveling duration. Thus, simple linear regression suggested that crowding was associated with a shorter orthodontic treatment duration.

The multivariate linear regression analysis performed thereafter indicated that horizontal anterior retraction was the only clinical variable significantly associated with orthodontic treatment duration. These results are consistent with the lack of consensus between previous studies on clinical factors affecting orthodontic treatment duration. Conflicting results have been reported regarding the effects of age, sex, and Angle classification on treatment duration^2,4,10,20^. There have also been discrepancies on the association of pretreatment ANB, FMA, PAR score, overjet, and overbite with treatment duration^7,11,21,22^. Although clinical parameters related to orthodontic treatment duration exhibited a low correlation in the current study, no conclusion can be drawn regarding the relationship of analyzed clinical parameters and treatment duration, as further clinical research is necessary.

Targeted next-generation sequencing revealed nine SNPs in six genes with a significantly different frequency between the extreme groups classified based on horizontal anterior retraction. The frequency of the C allele SNP of *WNT3A* (rs752107) was higher in the extreme long group compared with that in the extreme short group. Wnt signaling is crucial for the growth and maintenance of various tissues, including bone^23^. The WNT3A protein is particularly involved in the differentiation of mesenchymal stem cells (MSCs) into osteoblasts^24^. It plays an important role in bone remodeling and the maintenance of homeostasis by regulating bone formation through the inhibition of MSC differentiation into the osteoblast lineage under high intracellular WNT3A levels^25^.

Rs752107, as a 3′-UTR SNP, may contribute to an increase in bone formation through its effect on microRNA (miRNA) binding affinity. That is, it alters the binding site of miRNA, which normally inhibits *WNT3A* translation by binding the *WNT3A* mRNA transcript harboring the C allele. In contrast, the T allele may compromise miRNA binding and enhance WNT3A protein translation. Higher WNT3A protein levels would be expected to negatively regulate osteoblast proliferation and differentiation, in turn decreasing bone formation^25,26^. Thus, patients with the CC genotype would be expected to exhibit increased bone formation and higher bone density, consistent with the association observed in the present study^27^. Increased bone density may subsequently slow orthodontic tooth movement, prolonging treatment duration^12^.

There are few studies on the association between *WNT3A* genetic polymorphisms and orthodontic treatment duration. The current findings suggest the possibility of rs752107 being related to bone remodeling, tooth movement, and thus orthodontic treatment duration. However, further studies are required to elucidate the molecular mechanism through which this SNP regulates bone formation as well as to determine whether rs752107 is in linkage disequilibrium with other functional polymorphisms.

*SPP1* encodes OPN, which mediates alveolar bone resorption at the compression side when orthodontic force is loaded. In a recent study, OPN upregulation via ERK- and p38 MAPK-mediated signaling was reported at the tension side during orthodontic tooth movement^28^. OPN is associated with the shortening of orthodontic treatment duration via the acceleration of orthodontic tooth movement through the regulation of alveolar bone remodeling at both the compression and tension sides in response to orthodontic stress^19^.

Studies have previously reported the association between genetic polymorphisms of *SPP1* and bone density. The C allele of *SPP1* (rs4754) was suggested to be related to high bone density and low risk of fracture^29^, while the T allele of rs4754 was associated with low bone mineral density and a high risk of osteoporosis in women^30^. In the current study, the frequency of the CC genotype of *SPP1* SNPs (rs4754, rs9138) and the TT genotype of rs1126616 was high in the extreme long group. The C allele of rs4754 is associated with reduced *SPP1* function, resulting in decreased bone resorption and increased bone density. High bone density may slow the speed of orthodontic tooth movement and thus lengthen orthodontic treatment duration^12^.

The effects of other SNPs (rs1126616, rs9138) on bone biology and orthodontic tooth movement have not yet been studied, with the exception of a report on the significant relationship of rs9138 with external apical root resorption (EARR) in orthodontic treatment^31^. As a 3ʹ-UTR SNP, rs9138 can significantly affect miRNA-mediated *SPP1* degradation and its protein levels. Further research is required to assess the association of *SPP1* polymorphisms and orthodontic treatment duration.

We observed a high frequency of the GG genotype of the *SFRP2* SNP rs3810765 in the extreme long group. *SFRP2* encodes secreted frizzled-related protein-2 (sFRP-2), one of the sFRP family proteins that bind directly to Wnt proteins and appear to antagonize their effects by sequestering them from their receptor. However, the role of sFRP-2 in teeth, bone, and supporting tissues remains elusive^32^. sFRP-2 was reported to enhance the inflammatory response and inhibit bone formation^33^; however, another study showed that SFRP2 activates the osteogenic differentiation of apical papilla stem cells, increasing bone formation^34^. Although there is no research on the effect of rs3810765 in tooth and bone tissues, as a splice region SNP, rs381076 is expected to affect mRNA splicing, thereby altering RNA stability, and influencing *SFRP2* gene expression. Further molecular studies are necessary to determine how the G allele would contribute to longer orthodontic treatment duration.

The purinergic P2X7 receptor (P2RX7) is a member of the P2X subfamily of purinergic receptors activated by extracellular ATP. *P2RX7* expression has been reported in both osteoclasts and osteoblasts where it regulates bone formation and resorption. Further, P2RX7 was demonstrated to be involved in mechanically-induced signaling between osteoblasts and osteoclasts^35^. Loss of P2RX7 function leads to increased osteoclast numbers and decreased trabecular bone mass^36^. In *P2RX7* KO mice, alveolar bone formation was not significantly affected, but the susceptibility to external apical root resorption was increased with the application of orthodontic force^37^.

As a missense variant, *P2RX7* SNP (rs3751143) is related to various diseases of bones, teeth, and periodontal tissues. The C allele of rs3751143 might contribute to low bone density and a high risk of osteoporosis in postmenopausal women^38^. In the current study, the A allele frequency of rs3751143 was high in the extreme short group, whereas that of the C allele was not significantly different in the extreme long group. Nonetheless, no conclusion can be drawn regarding the association between rs3751143 and orthodontic treatment duration, as reliable results from a greater number of subjects as well as functional studies are required.

In this study, the frequency of the C allele of *TNFSF11* SNPs (rs12585229, rs931273) was significantly increased in the extreme long group, which might be associated with lower expression of the *TNFSF11* gene. *TNFSF11* encodes RANKL (receptor activator of NF-κB ligand), and *TNFRSF11A* encodes RANK (receptor activator of NF-κB). RANKL binds to its receptor RANK to induce the differentiation of osteoclasts through RANKL/RANK signaling^39^. During orthodontic tooth movement, the expression of *TNFSF11* is increased in osteocytes of the periodontal ligament where the orthodontic force is applied, and bone resorption occurs due to osteoclast activation via enhanced RANKL levels^40^. Thus, RANKL promotes orthodontic tooth movement by inducing bone resorption in the direction of tooth movement. When a drug formulation that slowly releases RANKL was injected into the mesial and distal areas of rat premolars, followed by the application of orthodontic force, faster tooth movement was observed^41^.

As the SNPs of *TNFSF11* (rs12585229, rs931273) and of *TNFRSF11A* (rs4524034) are intron variants, they are generally expected to have little effect on gene function. However, even an intron variant can affect gene expression at the transcription or translation level, as indicated by previous studies, wherein these SNPs were shown to be related to external apical root resorption^42^. While there have been some studies on genetic polymorphisms of *TNFSF11* that affect bone metabolism, no research on *TNFSF11* SNPs rs12585229 and rs931273 has been published. There is only a report relating the rs4524034 SNP of *TNFRSF11A* to menarche in women^43^. Nevertheless, the genetic polymorphisms of *TNFSF11* and *TNFRSF11A* are worth investigating in depth, considering their important role in orthodontic tooth movement.

This study has several limitations, including a small sample size, setting of the orthodontic treatment duration that excluded finishing period, and retrospective nature of the study. There were also practical difficulties encountered in the process of selecting patients suitable for the set criteria. In addition, this study did not elucidate the functional mechanism of how the SNPs are related to the treatment duration. Future studies are required to elucidate these mechanisms, validate the effect of the SNPs on the orthodontic treatment duration, and determine their influence in other cohort groups.

## Materials and methods

### Definition of orthodontic treatment duration

In this study, the duration of orthodontic treatment was defined as the leveling and anterior retraction period for space closing, as these processes are the most relevant to the duration of orthodontic treatment in clinical practice.

### Subjects

This retrospective study was approved by the institutional review board of Yonsei University Dental Hospital (number: 2-2016-0023). All subjects were Korean participants and unrelated. All clinical examinations were conducted in accordance with the Declaration of Helsinki, and written consent was obtained on paper with a description of the study prior to enrolling all subjects. Initially, 122 patients who received orthodontic treatment with maxillary premolar extractions at the Department of Orthodontics at Yonsei University Dental Hospital in Seoul, Korea from February 2008 to May 2020 were enrolled. Among them, 117 patients (24 males, 93 females, with an average age of 19.8 years) were finally selected; five patients were excluded owing to complications in the genetic analysis due to contamination, or because the patients could not proceed with the treatment due to personal reasons such as long-term absence, poor collaboration, and frequent breakage of brackets or appliances.

The selection criteria were as follows: (1) treatment with fixed orthodontic appliances in the permanent dentition, (2) maxillary premolar extracted on both sides and anterior teeth retracted for space closing, (3) panoramic and lateral cephalometric radiographs taken before and after orthodontic treatment, (4) no orthognathic surgery, (5) no systemic diseases affecting tooth movement, bone metabolism, or maxillofacial malformation.

All 117 patients were treated using the straight-wire appliance (SWA) technique, from a 0.016-in nickel-titanium to a 0.019 × 0.025-in stainless steel (G & H Orthodontics, Franklin, Ind.) with conventional brackets of 0.022 slots.

### Measurement of clinical parameters related to treatment duration

To determine the clinical parameters related to orthodontic treatment duration, 18 clinical variables of the subjects such as age, sex, Angle classification, horizontal anterior retraction, crowding, displacement, overjet, overbite, PAR score, ANB (T1: before treatment, T2: after treatment), FMA (T1, T2), and U1 to SN (T1, T2) were measured using pre-treatment dental models, lateral cephalometric radiographs, and chart review. Lateral cephalometric radiographs were acquired before and after orthodontic treatment by a trained radiographer using Cranex3 + (Soredex, Helsinki, Finland) at the Department of Oral and Maxillofacial Radiology, Yonsei University Dental Hospital. These lateral cephalometric radiographs were traced using V-Ceph™ 5.5 (Osstem, Seoul, South Korea).

**Figure 3:**
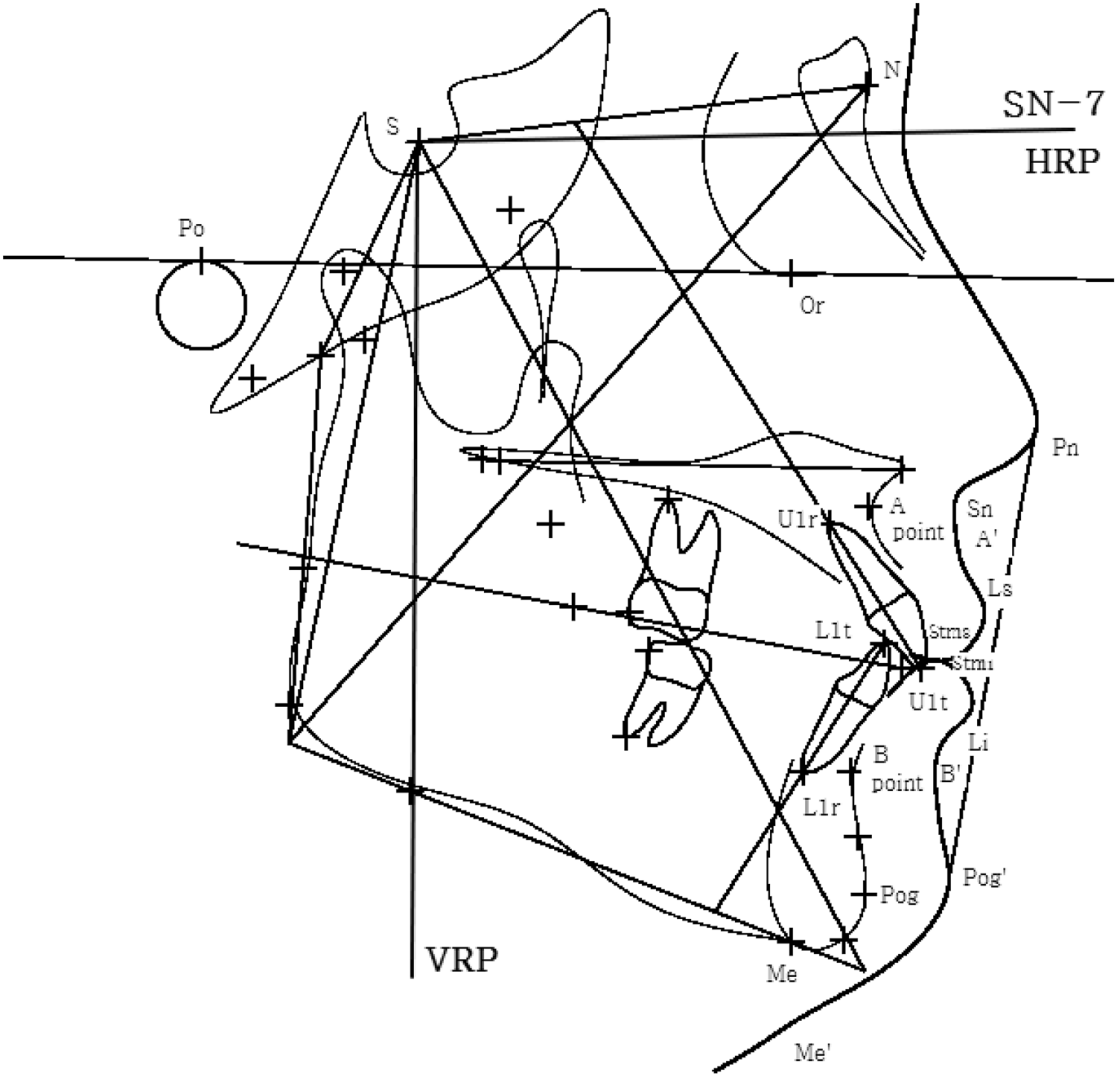
Reference lines and points for cephalometric analysis. Horizontal reference plane (HRP) is line minus 7° from the Sella–Nasion line. Individual landmarks are as follows: sella (S), nasion (N), A point (A), B point (B), pogonion (Pog), menton (Me), root apex of the maxillary central incisor (U1r), tip of the maxillary central incisor (U1t), tip of the mandibular central incisor (L1t), root apex of the mandibular central Incisor (L1r), pronasale (Pn), subnasale (Sn), soft tissue A point (A′), labrale superioris (Ls), stomion superioris (Stms), stomion inferioris (Stmi), labrale inferioris (Li), soft tissue B point (B′), soft tissue pogonion (Pog′), soft tissue menton (Me′).

Displacement, measured in mm, was defined as the sum of the absolute values of crowding and spacing to quantify abnormal tooth position. PAR score was obtained from the PAR index to quantify the degree of initial malocclusion^44^. Horizontal anterior retraction was defined as the difference between the tip of maxillary anterior teeth parallel to the HRP (horizontal reference plane) before (T1) and after (T2) orthodontic treatment in the lateral cephalometric radiograph, as measured in mm. HRP, proposed by Burstone^45^, was the line minus 7° from the Sella–Nasion line, and it was taken as the horizontal reference of orthodontic tooth movement in the study (Fig. 3).

### Reliability of the measurement method

Ten patients were randomly selected to check for errors in radiographic measurement, and twenty lateral cephalometric radiographs were acquired from them. Each radiograph was examined by the same operator after a 2-week interval to determine reliability of the measurement. The intraclass correlation coefficient (ICC) between the two examinations was over 0.9, indicating considerable consistency. The difference between the first and second assessments was not significant.

### Genetic analysis

Genomic DNA was isolated from saliva using the Oragene DNA self-collection kit (Genotek, Ottawa, Ontario, Canada) and extracted with the prepIT-L2P DNA extraction kit (DNA Genotek, Ottawa, Ontario, Canada) according to the manufacturer’s recommendations. DNA quantification was performed using Qubit.

For targeted next-generation sequencing analysis, previously reported genes and their SNPs related to tooth movement or bone metabolism were screened after a literature review (Supplementary Table 4). Cytokine genes that induce the inflammatory response around periodontal ligament tissues were selected as genes related to tooth movement. Genes involved in Wnt signaling and the RANK/RANKL pathway were also selected owing to their influence on orthodontic tooth movement and regulation of alveolar bone formation or resorption. Genes included only the coding sequence (CDS) region. For SNPs outside the CDS region, additional target probes were designed. Hybridization capture-based next-generation sequencing was employed, and sequencing data were obtained using a genome analysis tool kit. Sequencing reads obtained from Illumina NextSeq 500 platforms were further analyzed using BWA-MEM, Picard (v1.115), and Samtools (v1.1), and GATK (v4.0.4.0) was used to call single nucleotide variants.

### Statistical analysis

Statistical analyses were performed using R 3.5.2 (R Foundation) and GraphPad Prism software (GraphPad Inc., La Jolla, CA, USA). Univariate and multivariate linear regression were performed to analyze the correlation between parameters and orthodontic treatment duration. Differences in clinical parameters between extreme long group (showing long treatment duration) and extreme short group (showing short treatment duration) were analyzed using the χ^2^ test or *t*-test, as appropriate. The dominant, codominant, and recessive model was used in genetic association analysis. Genotype frequencies of each SNP were tested using the Hardy–Weinberg equilibrium. All P-values were based on two-sided comparisons, and P < 0.05 was considered statistically significant. The degree of linkage disequilibrium (LD) of *SPP1* and *TNFSF11* SNPs was examined using Haploview 4.2 software.

## Supplementary Information

Supplementary Tables.

## Data Availability

The accession number for the SRA data is PRJNA735789.

## Abbreviations

NGS: Next-generation sequencing
SNP: Single nucleotide polymorphism
SWA technique: Straight-wire appliance technique
PAR score: Peer assessment rating score
ANB: Angle of A point-Nasion-B point which denotes the relative position of the maxilla and mandible to each other
FMA: Frankfort-mandibular plane angle
U1 to SN: Angle of upper incisor to Sella-Nasion line
HRP: Horizontal reference plane
