# Supplementary material for: The effect of genetic polymorphisms on treatment duration following premolar extraction
Source: Sci Rep. 2021 Aug 5;11:15942. doi: 10.1038/s41598-021-94979-8 (PMC8342496; doi:10.1038/s41598-021-94979-8)
Supplement: Supplementary file 1 — Supplementary Tables. [file 41598_2021_94979_MOESM1_ESM.docx]

**The effect of genetic polymorphisms on treatment duration following premolar extraction**

Jiyon Yu^1^, Yoon Jeong Choi^1^, Sung-Hwan Choi^1^, Han-Sung Jung^2^, Ji Hyun Lee^3^*, Jung-Yul Cha^1^*

^1^Department of Orthodontics, Yonsei University College of Dentistry, Seodaemoon-gu, Seoul, Korea

^2^Division in Anatomy and Developmental Biology, Department of Oral Biology, Yonsei University College of Dentistry, Seodaemoon-gu, Seoul, Korea

^3^Department of Clinical Pharmacology and Therapeutics, Kyung Hee University College of Medicine, Dongdaemoon-gu, Seoul, Korea

***Co-corresponding authors; both authors contributed equally:

Jung-Yul Cha, Department of Orthodontics, Yonsei University College of Dentistry, Institute of craniofacial deformities, 50-1 Yonseiro, Seodaemun-gu, Seoul 03722, Korea; e-mail: jungcha@yuhs.ac

Ji Hyun Lee, Department of Clinical Pharmacology and Therapeutics, Kyung Hee University, College of Medicine, Dongdaemoon-gu, Seoul 02453, Korea, e-mail: hyunihyuni@khu.ac.kr

**Supplementary Table 1.** Genetic association with orthodontic treatment duration in all subjects for significant SNPs of extreme phenotype sampling study.

| ***Gene*** | ***SNP*** | ***Ref > Alt*** | ***Model*** | ***Beta (95%CI)*** | ***P value*** |
| --- | --- | --- | --- | --- | --- |
| *WNT3A* | rs752107 | T>C | Additive | 0.01165 (-0.002322 - 0.02562) | 0.1014 |
|  |  |  | Dominant | 0.004399 (-0.001086 - 0.009883) | 0.1149 |
|  |  |  | Recessive | 0.007249 (-0.003996 - 0.01849) | 0.2042 |
| *SPP1* | rs4754 | T>C | Additive | 0.01303 (-0.002230 - 0.02828) | 0.0935 |
|  |  |  | Dominant | -0.0004809 (-0.007303 - 0.006341) | 0.8892 |
|  |  |  | Recessive | 0.01351 (0.002163 - 0.02485) | **0.02** |
| *SPP1* | rs1126616 | C>T | Additive | 0.01303 (-0.002230 - 0.02828) | 0.0935 |
|  |  |  | Dominant | -0.0004809 (-0.007303 - 0.006341) | 0.8892 |
|  |  |  | Recessive | 0.01351 (0.002163 - 0.02485) | **0.02** |
| *SPP1* | rs9138 | A>C | Additive | 0.01113 (-0.005220 - 0.02748) | 0.1802 |
|  |  |  | Dominant | -0.001527 (-0.009337 - 0.006283) | 0.6993 |
|  |  |  | Recessive | 0.01265 (0.001224 - 0.02409) | **0.0303** |
| *SFRP2* | rs3810765 | C>T | Additive | -0.01125 (-0.02744 - 0.004948) | 0.1716 |
|  |  |  | Dominant | -0.006048 (-0.01686 - 0.004765) | 0.2702 |
|  |  |  | Recessive | -0.005199 (-0.01428 - 0.003885) | 0.2593 |
| *P2RX7* | rs3751143 | A>C | Additive | 0.003763 (-0.01098 - 0.01851) | 0.6141 |
|  |  |  | Dominant | 0.005174 (-0.006475 - 0.01682) | 0.3808 |
|  |  |  | Recessive | -0.001411 (-0.007635 - 0.004813) | 0.6542 |
| *TNFSF11* | rs12585229 | C>T | Additive | -0.01369 (-0.02994 - 0.002550) | 0.0977 |
|  |  |  | Dominant | -0.01139 (-0.02241 - -0.0003799) | **0.0427** |
|  |  |  | Recessive | -0.002299 (-0.01109 - 0.006491) | 0.6054 |
| *TNFSF11* | rs931273 | C>T | Additive | -0.01572 (-0.03208 - 0.0006327) | 0.0594 |
|  |  |  | Dominant | -0.01342 (-0.02447 - -0.002375) | **0.0177** |
|  |  |  | Recessive | -0.002299 (-0.01109 - 0.006491) | 0.6054 |
| *TNFRSF11A* | rs4524034 | A>G | Additive | -0.006944 (-0.02356 - 0.009671) | 0.4095 |
|  |  |  | Dominant | -0.01036 (-0.01989 - -0.0008305) | **0.0334** |
|  |  |  | Recessive | 0.003416 (-0.007180 - 0.01401) | 0.5244 |

Simple linear regression analysis was used.

Bold values denote statistical significance at the p < 0.05 level.

CI, Confidence interval

**Supplementary Table 2.** Predictive ability of SNPs and other clinical parameters for orthodontic treatment duration

| ***Parameters*** | ***Beta (95%CI)*** | ***P value*** |
| --- | --- | --- |
| Horizontal anterior retraction | 0.4612 (-0.1171 - 1.039) | 0.1169 |
| Crowding | -0.1903 (-1.294 - 0.9137) | 0.7333 |
| displacement | -0.135 (-1.308 - 1.038) | 0.82 |
| Presence of CC Genotype for rs9138 | 2.918 (0.1756 - 5.661) | **0.0372** |
| Presence of CC Genotype for rs931273 | 2.412 (-0.4825 - 5.307) | 0.1015 |
| Presence of AA Genotype for rs4524034 | 3.445 (0.09105 - 6.799) | **0.0442** |

The rs9138 of *SPP1* and rs931273 of *TNFSF11* were chosen in multivariate regression analysis for clinical and genetic variable, since three SNPs of *SPP1* (rs4754, rs1126616 and rs9138) and two SNPs of *TNFSF11* (rs12585229 and rs931273) were linked together, respectively.

Bold values denote statistical significance at the p < 0.05 level.

CI, Confidence interval

**Supplementary Table 3**. Genetic information of significant SNPs in extreme phenotype sampling study and allele frequency in all subjects

| Gene | SNP | Type of Variant | Allele Frequency |
| --- | --- | --- | --- |
| *WNT3A* | rs752107 | Intron Variant | T=0.22, C=0.78 |
| *SPP1* | rs4754 | Synonymous Variant | T=0.27, C=0.73 |
| *SPP1* | rs1126616 | Synonymous Variant | C=0.27, T=0.73 |
| *SPP1* | rs9138 | 3'-UTR Variant | A=0.30, C=0.70 |
| *SFRP2* | rs3810765 | Intron Variant | C=0.56, T=0.44 |
| *P2RX7* | rs3751143 | Missense Variant | A=0.71, C=0.29 |
| *TNFSF11* | rs12585229 | Intron Variant | C=0.59, T=0.41 |
| *TNFSF11* | rs931273 | Intron Variant | C=0.60, T=0.40 |
| *TNFRSF11A* | rs4524034 | Intron Variant | A=0.47, G=0.53 |

**Supplementary Table 4.** List of targeted sequencing regions

| *Genes* | *Additional regions* |
| --- | --- |
| *ALPL* |  |
| *CASP1* | rs530537 (Intron) |
| *CASP5* | rs554344 (2KB Upstream) |
| *IL-17A* | rs2275913 (2KB Upstream) |
| *IL1A* | rs1800587 (5'-UTR) |
| *IL1B* |  |
| *IL1RN* |  |
| *IL-6* | rs1800796 (Intron) |
| *IL-8* |  |
| *IRAK1* |  |
| *LRP1* |  |
| *LRP5* |  |
| *LRP6* |  |
| *DKK1* |  |
| *DKK2* |  |
| *DKK3* |  |
| *FRZB* |  |
| *FZD7* |  |
| *SFRP1* | rs16890444 (Intron) |
| *SFRP2* | rs3242 (3' UTR) |
| *SFRP4* |  |
| *SFRP5* |  |
| *WIF1* |  |
| *WISP3* |  |
| *WNT10B* |  |
| *WNT3A* | rs4653533 (Intron), rs752107 (Intron) |
| *WNT7B* |  |
| *SOST* | rs1230399 (Upstream), rs851054 (2KB Upstream), rs851056 (2KB Upstream) |
| *SPP1* | rs11730582 (2KB Upstream), rs9138 (3’UTR region), rs1126616, rs4754 |
| *TNF* | rs1800629 (2KB Upstream) |
| *TNFRSF11A* | rs12455775 (Intron), rs12956925 (Intron), rs12959396 (Intron), rs12970081 (Intron), rs17069845 (Intron), rs17069898 (Intron), rs17069902 (Intron), rs17069904 (Intron), rs17720953 (Intron), rs3826620 (Intron), rs4426449 (Intron), rs4485469 (Intron), rs4500848 (Intron), rs4524034 (Intron), rs4941125 (Intron), rs4941129 (Intron), rs6567272 (Intron), rs7233197 (Intron), rs7236060 (Intron), rs7237982 (Intron), rs7239667 (Intron), rs8083511 (Intron), rs8086340 (Intron), rs8089829 (Intron), rs8099222 (Intron), rs9951012 (Intron) |
| *TNFRSF11B* | rs3102735 (2KB Upstream), rs1032128 (Intron), rs11573856 (Intron), rs11573884 (Intron), rs11573901 (Intron), rs11573938 (Intron), rs1485289 (Intron), rs2875845 (Intron), rs3102724 (Intron), rs3102728 (Intron), rs3134057 (Intron), rs3134060 (Intron), rs7010267 (Intron) |
| *TNFSF11* | rs1038434 (Intron), rs12585229 (Intron), rs3742257 (Intron), rs931273 (Intron) |
| *VDR* |  |
| *P2RX7* |  |
